# Reliable Communication in Distributed Photovoltaic Sensor Networks: A Large Language Model-Driven Approach

**DOI:** 10.3390/s26030838

**Published:** 2026-01-27

**Authors:** Wu Dong, Xu Liu, Qing Liu, Guanghui Zhang, Ji Shi, Xun Zhao, Zhongming Lei, Wei Wang

**Affiliations:** 1 Power Dispatch Control Center, Guizhou Power Grid Co., Ltd., Guiyang 550000, China; dongwu@gz.csg.cn (W.D.); liuxu@gz.csg.cn (X.L.); liuging1949@sina.com (Q.L.); zgh199509@163.com (G.Z.); shiji2008.happy@163.com (J.S.); 15285123639@163.com (X.Z.); 2 School of Computer Science, Wuhan University, Wuhan 430072, China; zhongminglei@whu.edu.cn; 3 School of Electronic Information and Communications, Huazhong University of Science and Technology, Wuhan 430074, China

**Keywords:** distributed photovoltaic system, fault diagnosis, wireless transmission

## Abstract

Distributed photovoltaic (DPV) systems present a cost-effective and sustainable industrial energy solution, yet their reliable monitoring faces significant technological constraints. This paper proposes a hierarchical optimization framework that integrates hysteresis-based traffic shaping at the network layer with Large Language Model (LLM)-driven diagnostics at the application layer. The proposed dynamic algorithm minimizes latency and downtime by prioritizing critical fault data. Priority-based scheduling ensures this critical data is transmitted preferentially over routine sensor readings. At the application layer, the system utilizes physics-informed prompt engineering to perform zero-shot root cause analysis, circumventing the training data requirements of traditional classifiers. Under a 10 Mbps gateway bandwidth, our method achieves a 46.08% to 49.87% reduction in P50 latency compared to traditional approaches. Moreover, the LLM-powered diagnostic system provides detailed assessments, enabling precise fault diagnosis for DPV systems.

## 1. Introduction

**Figure 1:**
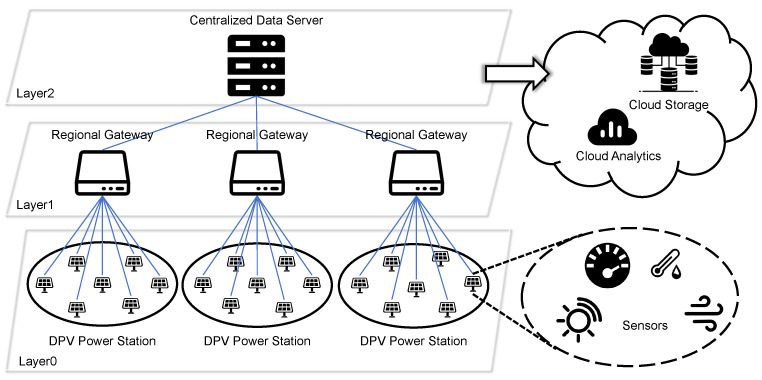
The architecture of our DPV monitoring network is a three-layer hierarchical system organized in a star topology. Layer 0 forms the base, consisting of numerous photovoltaic nodes equipped with various sensors to gather raw data. At the next level, Layer 1 is populated by regional gateways, each responsible for collecting data from a local group of photovoltaic nodes. Finally, all data converges at Layer 2, the central data server, which aggregates the information from all gateways for comprehensive analysis and management.

Distributed photovoltaic (DPV) systems are emerging as a cornerstone for cost-effective and sustainable energy solutions within diverse industrial applications. This trend aligns with broader energy forecasts, which project that solar photovoltaics will constitute the primary renewable energy source by 2050, satisfying at least 25% of the total global electricity demand [1]. Consequently, there is a significant impetus from both industrial and academic sectors to develop advanced monitoring frameworks for large-scale DPV installations (see Figure 1). These frameworks are designed to leverage reliable data analytics and intelligent sensor networks to streamline operational oversight. The principal objectives are to minimize technical complexity and operational expenditures, while simultaneously supporting critical energy management functions such as grid synchronization, efficiency enhancement, and the implementation of condition-based maintenance protocols.

However, achieving this level of operational reliability is often compromised by the system’s reliance on a conventional polling mechanism. As an inherently pull-based architecture, polling compels a central station to sequentially query every distributed sensor node, irrespective of whether a significant event has occurred. While simple, this rigid approach is inefficient for modern, large-scale deployments. Research into advanced smart grids highlights that such a request-driven method generates substantial communication overhead and is fundamentally unresponsive to asynchronous critical events, like system faults [2]. This inefficiency creates a compounding problem of sequential latency accumulation: as the number of monitored nodes grows, the polling cycle lengthens linearly. Consequently, a sensor signaling a critical fault may be forced to wait for an entire, often lengthy, sequence to complete before its data can be collected. This constitutes a major bottleneck for timely fault detection, a limitation that persists even under ideal communication conditions.

This inherent weakness of polling is severely compounded in typical DPV environments, where unreliable wireless communication links present a major obstacle. A primary factor is the strong electromagnetic interference (EMI) generated by the system’s power electronics [3]. Specifically, high-frequency switching in DC-DC converters and DC-AC inverters, a cornerstone of photovoltaic systems, inevitably produces significant EMI [4,5,6,7]. This high-frequency noise is not confined to the power converters themselves [8]; it propagates as conducted emissions along the extensive DC and AC cabling found in both utility-scale and residential installations [9]. Critically, this network of cables and the photovoltaic panels can act as unintentional antennas, radiating electromagnetic disturbances over a wide frequency range (from 150 kHz to over 1 GHz) that directly overlaps with wireless communication bands [8,10]. This EMI, combined with physical-layer challenges like signal attenuation and limited bandwidth, leads to significant packet loss and unpredictable transmission delays [9]. When these physical-layer instabilities are superimposed on the rigid, timing-dependent polling mechanism, the effect is devastating. This synergy, an inefficient protocol operating on an unreliable medium, drastically degrades data transmission efficiency, undermining the system’s ability to collect timely and complete data.

Reliable DPV monitoring is essential for operational efficiency and grid synchronization [11,12,13,14,15], and while various methodologies have been explored [16], each has distinct limitations. For instance, virtual collection techniques require high-fidelity reference data [17], while hardware-centric approaches using wireless sensor networks (WSNs) [18] or Internet of Things (IoT) architectures [19] suffer from high costs, maintenance burdens, and scalability issues. The quest to overcome these hurdles reflects a broader movement within IoT research toward creating more efficient and ubiquitous sensing systems. This includes innovations such as enabling communication between heterogeneous devices [20], securing networks with physical-layer properties [21,22], developing ultra-low-power connectivity via backscatter [23,24], and even harnessing ambient signals like GPS for passive sensing [25].

While the aforementioned advancements are notable, they have yet to close the significant gap between existing tools and the demands of truly reliable, large-scale DPV monitoring. At the heart of this challenge lie the inherent limitations of conventional RF wireless communications, characterized by non-adaptive polling, bandwidth inefficiency, and susceptibility to interference. The inevitable outcome is the delayed or lost transmission of fault-critical data, a failure that directly threatens system reliability and grid stability.

This paper puts forth the thesis that a viable solution must adopt a holistic, multi-layered architecture. We contend that such a framework is necessary to systematically rectify deficiencies across the system’s primary strata: (1) sequential latency accumulation at the perception layer, (2) bandwidth saturation at the network layer, and (3) the prohibitive labor costs and cognitive bottlenecks of human-centric analysis at the application layer.

The emergence of large language models (LLMs) presents a paradigm shift for addressing these limitations. With demonstrated capabilities across diverse applications [26,27,28], LLMs possess robust contextual understanding, pattern recognition, and logical reasoning abilities. Crucially, this approach fundamentally differs from classical machine learning (ML) algorithms, such as Support Vector Machines (SVMs) or Random Forests, which are inherently interpolative and require large-scale labeled datasets for supervised training. Classical ML models excel at classifying known fault types, but struggle to generalize to novel unseen anomalies the long-tail challenge, and lack the capacity to deliver interpretable human-readable diagnostic reasoning. In contrast, an LLM-driven agent can perform extrapolative zero-shot diagnosis by capitalizing on its extensive pre-trained knowledge base to reason from first principles, synthesizing heterogeneous data sources such as numerical sensor streams, textual weather logs, and static panel specifications through a unified semantic framework. These attributes enable the automation of intelligent diagnostics, thereby optimizing the fault detection capabilities within DPV systems.

**Figure 2:**
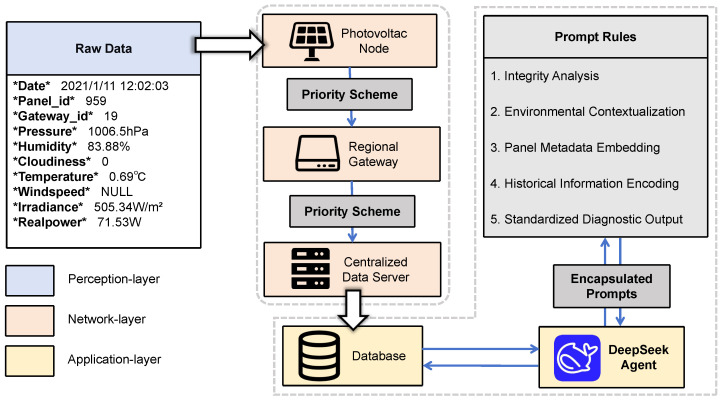
The proposed DPV monitoring system consists of three layers. At the perception layer, various sensors detect the status of photovoltaic nodes. At the network layer, operational data from monitoring devices in the perception layer is transmitted through regional gateways according to the priority scheme, ultimately reaching the centralised data server. At the application layer, the Deepseek Agent (https://api-docs.deepseek.com/zh-cn/api/deepseek-api/, accessed on 25 December 2025) encapsulates prompts according to specific prompt rules and performs fault diagnosis on database data.

To address the multifaceted challenges of reliable DPV monitoring, this paper proposes a three-tiered framework, as illustrated in Figure 2. The primary technical contributions and their distinctions from the existing literature are summarized as follows:

1. We propose a novel dynamic status labeling mechanism that functions as an active control signal for network resources. Distinct from traditional static threshold or event-triggered methods susceptible to priority oscillation namely flapping under environmental noise, our approach incorporates a hysteresis-based state locking mechanism. By enforcing temporal stability constraints on status transitions, this method effectively mitigates the impact of intermittent interference and facilitates cross-layer optimization of the communication protocol.
2. We introduce a priority-based scheduling strategy that fundamentally differs from standard MAC-layer Quality of Service (QoS) or edge-offloading frameworks. While traditional methods focus on reordering packets after they have been queued, namely congestion management, our algorithm implements source-side traffic shaping. By dynamically suppressing the data generation of normal nodes based on their semantic status, the system preemptively prevents bandwidth saturation, achieving a 46.08% to 49.87% reduction in P50 latency for critical data in bandwidth-constrained scenarios.
3. We integrate a domain-specialized LLM agent to replace conventional supervised classifiers. Unlike these black box models that depend on large-scale labeled datasets and face challenges with unseen fault types, our agent leverages zero-shot semantic reasoning. By synthesizing environmental context, physical metadata, and historical trends, the system automates root cause analysis for long-tail untrained fault scenarios, delivering actionable interpretable maintenance insights that bridge the divide between signal detection and operational decision-making.

The remainder of this paper is organized as follows: Section 2 provides an overview of relevant background knowledge on DPV monitoring systems. Section 3 details the design of our proposed system, including its priority scheduling strategy and LLM-based diagnostic mechanism. In Section 4, we describe our simulation, covering the data preprocessing approach, simulator design, and the configuration of comparative experiments. Section 5 presents a comprehensive evaluation of the system’s performance. Finally, Section 6 concludes the paper with a summary of our findings and potential directions for future work.

## 2. DPV Monitoring Network Primer

### 2.1. DPV Monitoring Network Architecture

**Figure 3:**
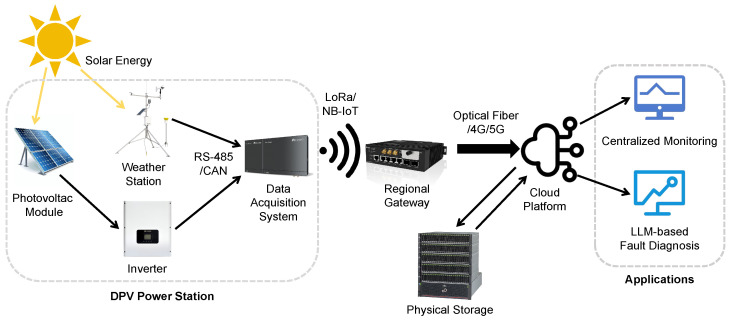
End-to-end system architecture for DPV monitoring and fault diagnosis.

The DPV monitoring network constitutes a sophisticated cyber–physical framework that integrates intelligent sensing devices, secure data transmission pathways, and cloud-based analytical engines [29]. This multilayered architecture enables continuous operational oversight of geographically dispersed photovoltaic installations. It achieves this through synchronized coordination between edge-level data acquisition nodes, intermediate communication gateways, and centralized cloud platforms. The system facilitates reliable anomaly detection, performance benchmarking, and predictive maintenance strategies while maintaining robust data integrity across heterogeneous network environments. The architecture of a typical DPV monitoring network comprises several key components (see Figure 3):

**Photovoltaic module.** This is the fundamental energy-generating unit. Each module contains a photovoltaic panel that captures solar irradiance and converts it directly into direct current electricity.

**Weather station.** A dedicated weather station, equipped with a suite of environmental sensors, is deployed to continuously monitor local meteorological conditions at the DPV site. This provides critical data points such as ambient temperature, humidity, air pressure, wind speed, and solar irradiance, which are essential for performance modeling and diagnostics.

**Inverter.** The inverter is a critical power electronics component that converts the DC electricity generated by the photovoltaic modules into high-voltage alternating current electricity, making it suitable for injection into the utility grid or for local consumption.

**Data acquisition system (DAS).** The DAS serves as the primary data collection hub at the local level. It is responsible for gathering a wide array of operational and environmental data from sources like the inverters and the weather station. This includes electrical parameters (power output, voltage, current), environmental metrics, and equipment status information.

**Regional gateway.** This component acts as an intermediary aggregation point. The gateway collects and performs preliminary processing on data from multiple distributed DASes before securely transmitting the aggregated information to the central cloud platform. To accommodate the specific operational requirements of DPV environments, the system assumes a heterogeneous communication protocol stack where the local link between photovoltaic nodes and the gateway utilizes Low-Power Wide-Area Network (LPWAN) standards to ensure robust long-range connectivity amidst electromagnetic noise, while the backhaul link from the gateway to the cloud relies on high-speed cellular networks. This dual-protocol architecture allows the system to balance the low-power constraints of edge sensors with the high-bandwidth requirements of the centralized diagnostic server.

**Cloud platform.** The cloud platform represents the centralized intelligence and control center of the entire DPV network. It serves as the primary repository for long-term historical data storage and hosts the advanced LLM agents responsible for executing the fault diagnosis logic described in Section 3.

**Physical storage.** Local physical storage provides critical redundancy and enhances system responsiveness. It serves to back up data from the DAS and buffers sensor readings locally to prevent data loss during network outages, ensuring continuity for the historical sliding-window analysis.

### 2.2. Data Volume and Transmission in DPV Monitoring Networks

The data volume generated by DPV monitoring networks is substantial and grows in direct proportion to the expansion of photovoltaic installations. To ensure accurate monitoring and control, each data acquisition node collects a multitude of parameters sampled at high frequencies, contributing to this large and continuous data stream.

**Data Transmission Process.** The data transmission process within a DPV monitoring network is a two-stage hierarchical flow. In the first stage, data is transmitted from the distributed photovoltaic nodes at the edge to their designated regional gateway. In this capacity, the gateway acts as an intermediary aggregation point for multiple nodes. In the second stage, the gateway forwards the aggregated data to the centralized Data Server. This transmission may be preceded by local data processing or analysis at the gateway level.

**Architectural Assumptions.** In this paper, we make several simplifying assumptions to facilitate the design of our scheduling algorithms. We assume that edge photovoltaic nodes cannot send data to each other directly, a constraint that holds for most contemporary DPV systems, as they are designed to transmit data only to designated gateways. Similarly, we assume that gateways cannot send data to each other, as this would consume bandwidth that could otherwise be used for server communication and is not part of standard system designs. Finally, we assume that devices operating at the same hierarchical level function independently. While these assumptions are foundational to our current model, we acknowledge that relaxing them could be a valuable avenue for future work.

## 3. Methodology

### 3.1. System Architecture

The proposed DPV monitoring system is composed of four core components: distributed photovoltaic nodes, regional gateways, a centralized data server, and a DeepSeek-based diagnostic module. This architecture is designed to simulate large-scale DPV deployments, featuring reliable anomaly detection and prioritized data transmission.

**Distributed Photovoltaic Nodes.** In the simulation, each node is responsible for generating sensor data streams that emulate real-world conditions, including barometric pressure, humidity, cloud coverage, temperature, wind speed, irradiance, and real power. These data points are generated at predetermined intervals. A key function of each node is to respond to polling requests from its designated regional gateway in a timely manner to ensure data is successfully transmitted to the next hierarchical layer.

**Regional Gateways.** Each regional gateway manages a dedicated cluster of photovoltaic nodes, with non-overlapping management areas. During the simulation, a gateway’s primary responsibilities are twofold: first, to poll the photovoltaic nodes within its jurisdiction to acquire reliable monitoring information, and second, to process polling requests from the centralized data server. This ensures the timely transmission of collected data to the application layer for analysis.

**Centralized Data Server.** The centralized data server orchestrates the system-wide monitoring process by polling each regional gateway in a cyclic manner. Upon receiving data from the gateways, the server archives it in a file system. If an anomaly is detected within the incoming data, the server forwards the relevant information to the DeepSeek-based diagnostic module for detailed fault analysis.

**DeepSeek-based Diagnostic Module.** This module capitalizes on DeepSeek-Chat, a practical and open-source Large Language Model based on a Mixture-of-Experts (MoE) Transformer architecture. To ensure generalizability and minimize deployment costs, the system employs the model in its pre-trained frozen state, deliberately avoiding the need for domain-specific fine-tuning or weight updates. Instead, the module relies on the agent’s rigorous prompt engineering framework that comprises integrity analysis, environmental contextualization, and historical encoding to align the model’s general reasoning capabilities with the specific physical constraints of the photovoltaic domain. The agent establishes a dedicated session for each regional gateway, maintaining a unique dialogue context corresponding to the management area of the respective gateway and facilitating iterative multi-round interactions for in-depth fault diagnosis.

**Temporal Coordination Strategy.** To sustain system coherence under real-world clock constraints and variable transmission delays, the three layers achieve coordination through a source-synchronized timestamping protocol. We assume all distributed nodes are synchronized via the Network Time Protocol (NTP). Crucially, every data packet is marked with an immutable generation timestamp at the perception layer immediately upon sensing acting as the invariant reference throughout the entire data lifecycle. The network layer leverages it to uphold chronological ordering within the priority queues, thereby preventing out-of-order transmission amid congestion, while the application layer relies on it to execute precise temporal alignment. This alignment ensures sensor readings are correlated with the exact meteorological context, such as historical irradiance logs corresponding to their generation time, thus decoupling diagnostic accuracy from network-induced latency.

### 3.2. Dynamic Priority Scheduling Scheme

To enhance the responsiveness of the DPV monitoring system to anomalous data, we propose a dynamic, priority-based scheduling scheme. This scheme is founded on three core principles:

**Priority Queue.** In place of a conventional FIFO queue, we incorporate a hierarchical priority queueing mechanism at both distributed photovoltaic nodes and regional gateways. This data structure directly maps perception-layer status labels to network transmission slots through a strict priority discipline. Specifically, the queue sorts pending transmission requests based on a compound key with the primary sorting criterion being the status label which designates anomalous as the highest logical priority and the secondary criterion being the generation timestamp. This ensures that any data designated as anomalous effectively bypasses all normal routine traffic in the buffer regardless of its arrival time. Consequently, the scheduler is required to transmit all critical fault data completely before initiating the transmission of routine sensor readings, thereby significantly reducing latency for high-value diagnostic information.

**Dynamic Status Labeling.** Each regional gateway incorporates a hysteresis-based dynamic status labeling mechanism to regulate the polling priority of its subordinate photovoltaic nodes. A node’s status is determined through an asymmetric state-transition logic engineered to prioritize fault responsiveness while alleviating system instability arising from intermittent electromagnetic interference. Specifically, the gateway performs real-time validity verification of each incoming data packet by confirming whether a sensor reading contains NULL values or violates predefined physical operating ranges such as voltage or current exceeding safety limits, in which case the node’s status is immediately designated as anomalous. This ensures an immediate response to potential faults. However, reversion to a normal state is governed by a stability verification protocol that requires an anomalous node to be reclassified as normal only after it successfully transmits valid data for three consecutive polling cycles. This design embeds the principle of control hysteresis to mitigate priority oscillation, a phenomenon in which a node frequently fluctuates between states due to transient environmental noise. Our sensitivity analysis in Section 5.4 confirms that requiring three consecutive valid data transmissions as the threshold achieves the optimal balance between responsiveness and resource efficiency; a lower number of required cycles risks prematurely downgrading the priority of nodes with intermittent faults, while a higher number results in unnecessary bandwidth occupancy by healthy nodes. Based on this status, the gateway dynamically reconfigures its scheduling by polling anomalous nodes in every cycle to ensure high-resolution monitoring, whereas normal nodes are polled at a reduced frequency of once every three cycles to reserve bandwidth for critical data streams. To ensure a comprehensive baseline assessment upon system startup, all photovoltaic nodes are initialized to the anomalous state.

**Greedy Transmission Strategy.** The data transmission process employs a modified greedy strategy that differs fundamentally from standard MAC-layer QoS protocols. While standard QoS mechanisms, such as priority tagging or guaranteed time slots, focus on reordering packets already in the transmission queue, our strategy implements content-aware traffic shaping at the network layer to regulate traffic generation itself. Under this scheme, the gateway prioritizes polling of anomalous nodes in every cycle, striving to leverage maximum available bandwidth to transmit critical data to the server. Normal nodes are only polled when residual bandwidth is available after satisfying all anomalous data transmission demands or during their mandatory check-in cycles. This approach differs from physical-layer prioritization in its role as a source-side filter; rather than merely managing channel contention across all nodes, it proactively suppresses data generation from healthy nodes. This ensures that in bandwidth-constrained environments, channel capacity is not occupied by routine low-value telemetry but is instead dedicated to guaranteeing the real-time delivery of fault-critical information.

**Figure 4:**
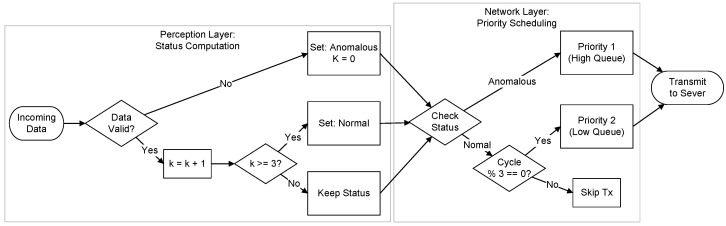
A block diagram of the dynamic priority scheduling algorithm. The flowchart details the validity check and hysteresis logic within the perception layer (**left**) and the resulting conditional scheduling executed at the network layer (**right**).

To summarize the interaction between these components, the complete algorithmic workflow is visualized in Figure 4. This formal block diagram explicitly maps the data flow from signal acquisition to transmission, illustrating the control dependencies between the perception-layer hysteresis counter (k) and the network-layer scheduling decision. As depicted, the timing of transmission events is strictly coupled to the derived status, ensuring that network resources are dynamically allocated based on the semantic urgency of the data.

### 3.3. Zero-Shot Adaptation Mechanisms

To mitigate the limitations of traditional supervised learning models in dynamic industrial environments, our architecture represents a fundamental departure from the standard classification paradigm adopted by CNNs or GNNs. While such data-driven models achieve high accuracy on known fault patterns, they function as black boxes that lack interpretability and demand extensive retraining when encountering novel fault types or evolving environmental conditions. In contrast, our approach capitalizes on an LLM to perform physics-informed semantic reasoning rather than statistical pattern matching. By treating sensor data not merely as numerical vectors but as contextualized information infused with physical significance, the system enables zero-shot diagnosis through leveraging the LLM’s extensive pre-trained knowledge base. This allows the agent to deduce the root causes of unseen anomalies through logical synthesis of meteorological context, panel metadata, and historical trends, thereby delivering interpretable and actionable insights that transcend the rigid class labels of conventional classifiers.

In the proposed system, each regional gateway is supported by a dedicated DeepSeek diagnostic agent operating at the application layer of the centralized data server. These agents are tasked with interpreting sensor data from distributed photovoltaic nodes, identifying potential faults, and generating expert-level diagnostic reports. A fundamental component of the agent design is its capacity for zero-shot adaptation, enabling it to accurately diagnose novel fault types, adapt to new environmental contexts, and generalize across different panel configurations without requiring any task-specific supervised training.

To achieve this zero-shot adaptability, each agent employs a structured diagnostic pipeline composed of five interlinked components. By leveraging sophisticated prompt engineering, this pipeline simulates expert reasoning, transforming raw sensor data into a rich, interpretable diagnostic context. This structured context enables the DeepSeek LLM to perform complex inference via in-context learning. The five components and their roles in facilitating LLM reasoning and generalization are detailed below:

**Integrity Analysis.** The initial phase examines the completeness and integrity of incoming photovoltaic monitoring data with a specific focus on identifying missing timestamps or NULL sensor values resulting from network congestion or packet loss. This integrity verification is explicitly integrated into the prompt, guiding the LLM to prioritize attention on potential hardware failures or communication faults prior to conducting any performance calculations. Crucially, this step mitigates the risk of diagnostic errors arising from incomplete data by avoiding blind imputation of missing values which might result in hallucinations or false positives and instead framing the absence of data as a primary diagnostic signal in itself. This enables the LLM to perform zero-shot reasoning on fault probabilities, drawing on its general knowledge to differentiate between physical component malfunctions and transmission interruptions. For instance, consistent absence of wind speed data alongside subzero temperatures may indicate a frozen anemometer while sporadic gaps across all data channels would logically prompt the agent to diagnose a network layer congestion issue rather than a photovoltaic panel defect.

**Environmental Contextualization.** To facilitate the LLM in distinguishing between genuine hardware faults and external environmental constraints, a rule-based model establishes a physics-informed benchmark baseline for each diagnostic session. Through synthesizing the current time of day, season, ambient temperature, and cloud cover data, the system derives an expected irradiance level and a corresponding theoretical power output range based on standard photovoltaic models. These calculated baselines are integrated into the prompt as contextual anchors, effectively equipping the LLM with a dynamic healthy signature for the specific operating moment. Consequently, the LLM does not need to memorize static fault patterns; instead, it is tasked with identifying fault signatures through detecting and interpreting semantic discrepancies between the actual serialized sensor data and this physics-based expectation. For example, the model can logically deduce that a 25% power deficit constitutes a fault signature when the sky is clear, but a normal operational adjustment when the contextual anchor indicates heavy cloud cover, which is a distinction derived solely from contextual logic rather than prior training.

**Panel Metadata Embedding.** Each agent is initialized with static, a priori knowledge about the specific panel it supervises, including its surface area and nominal energy conversion efficiency. These physical constants are embedded directly into the prompt to ground the LLM’s understanding of the panel’s operational limits. For example, given a known conversion efficiency, the LLM can calculate the theoretical maximum power output from the measured irradiance and use this to determine if the actual power generation is anomalously low. This quantitative reasoning relies on the embedded metadata as a stable reference frame, allowing the model to generalize to panels with different specifications simply by adapting its calculations to newly embedded parameters.

**Historical Information Encoding.** To facilitate LLM in conducting longitudinal analysis on otherwise static data snapshots, the agent retrieves a sliding window consisting of the 15 most recent data records for the specific panel from local storage. Crucially, this raw numerical time-series data is converted via serialization into a structured textual representation which takes the form of a Markdown-formatted table prior to its injection into the prompt. This serialization addresses the modality gap between numerical sensor logs and the language-based model, transforming high-frequency sensor readings into an interpretable symbolic format capable of preserving temporal continuity. Through analyzing this structured historical data in conjunction with current readings, the LLM can infer complex temporal patterns that include gradual performance degradation, intermittent volatility and sudden step-changes, thereby enabling it to reliably distinguish between persistent hardware malfunctions and transient environmental variations.

**Standardized Diagnostic Output.** Finally, the agent enforces a structured response format, compelling the model to categorize findings into a defined taxonomy of detectable fault types. Specifically, the diagnostic scope is calibrated for SCADA-level telemetry and covers three primary categories: (1) Data Integrity Faults, including sensor freezing, calibration drift, and communication packet loss; (2) Environmental Anomalies, such as partial shading, snow accumulation, and soiling, which are semantically distinguished from hardware defects using meteorological context; and (3) Electrical Component Failures, including String Open-Circuits, Inverter Trips, and gradual Panel Degradation inferred from longitudinal historical trends. When encountering a novel fault type, this structured framework guides the model to systematically analyze the provided context (e.g., comparing historical data, checking metadata) and generate a coherent diagnosis. This output structure stabilizes the generalization process by imposing clear reasoning boundaries, promoting consistency and interpretability even for unfamiliar scenarios.

By integrating these five components, the DeepSeek diagnostic agent transforms unstructured and heterogeneous photovoltaic monitoring data into a structured, domain-aware prompt. This transformation enables a general-purpose LLM to perform highly specialized diagnostic tasks without any fine-tuning. The pipeline facilitates sophisticated reasoning by providing explicit context (metadata, historical trends, environmental baselines) that the LLM can logically interconnect to form robust conclusions. Crucially, it enables generalization by framing novel scenarios, including new fault types and unseen panel configurations, within a familiar analytical structure. This allows the model to apply in-context learning to adapt its reasoning on the fly. This design enables the rapid deployment of intelligent diagnostics across large-scale DPV systems, ensures consistency across diverse fault cases, and significantly lowers the barrier to entry for advanced analytics in distributed energy environments.

## 4. Simulation

To rigorously evaluate the scalability and robustness of the proposed architecture prior to physical deployment, we implement our system in a high-fidelity trace-driven simulation environment. While physical testbeds yield valuable hardware insights, they are often scale-limited and lack the flexibility to safely reproduce extreme failure scenarios, such as system-wide blackouts or massive EMI storms. We therefore adopt a custom discrete-event simulator designed to model the interactions among 5000 distributed nodes at a scale that aligns with real-world industrial demands. To ensure the empirical validity of the experiments, the simulator is driven by real-world photovoltaic monitoring traces sourced from the Shandong University Power System Economic Operation Team’s Energy & Meteorology Research Service Platform [30]. This approach combines the scalability of software simulation with the empirical realism of real field data.

### 4.1. Photovoltaic Monitoring Data

Our simulation framework is grounded in a publicly available benchmark dataset curated by the “Shandong University Power System Economic Dispatch” research group. This dataset provides an authentic foundation for our experiments, containing key environmental and operational variables from a real-world photovoltaic installation, including irradiance, atmospheric pressure, humidity, cloudiness, sunshine duration, and both theoretical and actual power output.

However, the source dataset is subject to two limitations that render it unsuitable for the purposes of this study. Firstly, it originates from a single photovoltaic node, and secondly, it has a coarse temporal resolution of 15 min. In order to address these issues, the initial procedure is to perform temporal upsampling. Linear interpolation is applied across all sensor channels to produce a time series with a 1-s resolution. The selection of this method was driven by the necessity to preserve the inherent temporal continuity of photovoltaic behaviour, whilst concurrently generating the high-granularity data that is essential for real-time simulation.

To simulate a distributed system comprised of multiple, independent nodes, we synthesize distinct data streams for each node. This is achieved by applying a unique, uniformly drawn random scaling factor from the range [0.95, 1.05] to the interpolated base series of each simulated panel. This process introduces plausible panel-level heterogeneity, simulating minor performance deviations arising from hardware differences, localized microclimates, or varying degrees of panel aging, thereby enriching the diversity of the simulated inputs.

Furthermore, to enhance the realism of the simulation, we incorporate a probabilistic sensor failure model. For each sensor channel on every photovoltaic node, a failure event is triggered if a pseudo-randomly generated value falls below a predefined failure threshold, resulting in the corresponding sensor value being marked as missing. To regulate the overall fault density and prevent unrealistic scenarios of cascading failures, this failure threshold is automatically halved for a given panel immediately after a fault has been introduced. This probabilistic fault injection mechanism strikes a balance between realism and data integrity, aiming to approximate typical fault rates observed in field deployments. Crucially, this mechanism acts as a functional abstraction of the intense EMI detailed in Section 1, effectively simulating the random data loss and signal degradation commonly observed in the perception layer of DPV systems.

The multi-stage data augmentation pipeline, incorporating temporal interpolation, randomised scaling, and controlled fault injection, transforms the original single-node dataset into a large-scale, high-resolution, and fault-aware dataset suitable for simulating realistic DPV networks. This augmented dataset constitutes the fundamental foundation of the proposed simulation framework. It is acknowledged that synthetic data is incapable of fully replicating the entire spectrum of complex, unpredictable fault patterns encountered in the field. However, this methodology effectively simulates the data characteristics and transmission challenges of large-scale DPV systems under common fault types. The environment thus provided is one of controlled yet representative evaluation, the purpose of which is to ensure that the observed performance trends and diagnostic capabilities align with practical operational demands.

### 4.2. DPV System Simulator

To evaluate the proposed architecture under realistic operational conditions, we developed a discrete-event simulation framework specifically tailored for DPV monitoring systems. The simulator is governed by a virtual time manager that advances the simulation in deterministic, 1-millisecond increments, enabling the capture of fine-grained interactions between system components.

The simulation is configured to model a massive, utility-scale deployment scenario comprising 5000 distributed photovoltaic nodes managed by 100 regional gateways. We select this high-volume configuration, rather than smaller setups of 50 or 500 nodes, to rigorously stress-test the scalability of the proposed architecture. The system scalability is underpinned by its hierarchical divide-and-conquer topology where each regional gateway is responsible for a dedicated zone of 50 nodes. This modular design ensures that the polling complexity remains constant at the edge regardless of the total network size. Consequently, the simulator can scale linearly to support tens of thousands of nodes simply by instantiating additional gateway objects, validating the framework applicability to both small industrial rooftops and large-scale solar farms.

At each simulation timestep, the framework processes the internal state transitions of all active components according to the following logic:

(i) Distributed Photovoltaic Nodes. Each node continuously generates monitoring data, including environmental and electrical parameters, at a high temporal resolution (1-s intervals). These data readings are buffered locally, awaiting a polling request from their corresponding gateway.

(ii) Regional Gateways. Gateways execute fixed polling schedules to collect data from their 50 associated nodes. Once collected, the data is queued and prepared for transmission to the centralized server for diagnostics and system-wide aggregation.

(iii) Transmission Modeling. For each data transfer, the simulator computes a realistic communication delay that integrates queueing latency and bandwidth-constrained serialization time. Regarding physical layer reliability, the simulation employs a payload corruption model to characterize environmental interference. Rather than simulating total packet loss in which the entire frame is dropped by the medium, we model the effects of EMI and sensor instability as intra packet data invalidation through probabilistic marking of specific sensor values within a successfully transmitted frame as NULL or invalid (as defined in Section 4.1). Under this model, packet retransmission is explicitly disabled. Since the data invalidity originates from the source capture stage, the perception layer rather than transmission corruption retransmitting the frame would not recover the missing information. The system thus accepts the partially corrupted packet to maintain the sampling timeline and relies on the subsequent polling cycle to acquire updated valid readings. Furthermore, due to the strict centralized polling schedule, packet collisions are structurally eliminated, obviating the requirement to model random backoff delays linked to CSMA/CA protocols.

The primary metric for assessing system responsiveness is end-to-end latency. This is defined as the total virtual time elapsed from the moment data is generated at a photovoltaic node to its successful reception by the central server. This metric holistically reflects the combined efficiency of the data collection, transmission, and queueing mechanisms across the system’s hierarchical layers.

By employing an event-driven simulation model with high-resolution timing, our framework facilitates a rigorous analysis of performance bottlenecks under varying data rates and gateway loads. This allows for a thorough evaluation of the architecture’s behavior under scale and delay pressures, thereby informing design decisions for practical, real-world deployments.

### 4.3. Baseline Comparison Schemes

To rigorously evaluate the performance of our proposed dynamic priority scheduling scheme, we benchmark it against two established data collection strategies that dominate the current landscape of industrial photovoltaic monitoring. These baselines represent the trade-off between deterministic fairness and maximum throughput.

**Conventional Polling Scheme.** This baseline models the standard, non-adaptive polling approach widely deployed in industrial SCADA systems. As noted in a recent comprehensive review of SCADA architectures [31], dominant industrial protocols such as Modbus, DNP3, and IEC 60870-5 fundamentally rely on this Master-Slave interrogation mechanism to ensure deterministic communication. In this scheme, both regional gateways and the central server operate on a strict cyclic schedule, querying photovoltaic nodes in a fixed sequential order and retrieving a limited quota of up to ten data records from each during every polling cycle. This method prioritizes fairness and predictability, ensuring equal bandwidth allocation for all nodes regardless of operational status, and serves as our primary reference for quantifying the latency penalties imposed by rigid, context-agnostic scheduling. This method serves as the primary reference for evaluating performance against the current industrial status quo.

**Conventional Greedy Scheme.** This baseline implements an aggressive bulk retrieval strategy designed to minimize buffer accumulation without semantic prioritization. Whenever a gateway polls a photovoltaic node, this approach attempts to collect the entire buffered data queue rather than a limited quota, with the central server similarly retrieving complete queues from each gateway during its polling round. This scheme simulates a best-effort, first-come-first-served strategy, establishing a performance benchmark for queue-draining capability and illustrating the latency characteristics of a system that maximizes raw throughput but lacks the intelligence to distinguish routine telemetry from critical fault alerts.

## 5. Evaluation

To ensure a fair and rigorous comparison, all three scheduling schemes were evaluated under identical, controlled simulation conditions. The simulation was configured to commence at a virtual time of 12:00:00 on 11 January 2021, and run for a simulated duration of one hour. To ensure a controlled evaluation environment, key network and data parameters are held constant across all experiments. The uplink transmission rate for each photovoltaic node is configured to 13.1 kbps. Crucially, each photovoltaic data packet is set to a uniform fixed size of 440 bits, regardless of whether the payload contains normal telemetry or anomalous fault signatures. This uniformity mirrors the fixed-frame structure of standard industrial protocols (e.g., Modbus) and ensures that any observed performance differentiation is driven strictly by the scheduling logic rather than variations in transmission serialization time. To assess performance under varying network constraints, the simulations were replicated across three distinct gateway uplink bandwidth settings: 50 Mbps, 10 Mbps, and 5 Mbps. The results of this comparative evaluation are presented in the following section.

### 5.1. End-to-End Latency

**Figure 5:**
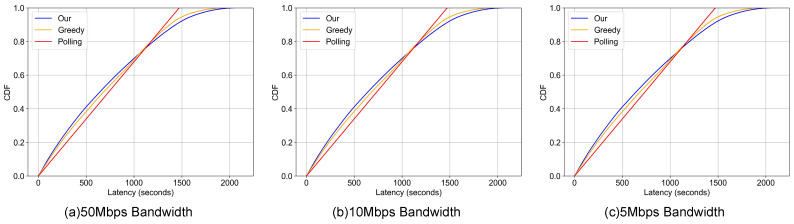
Overall End-to End-Latency Comparison. *For different gateway uplink bandwidth.*

**Figure 6:**
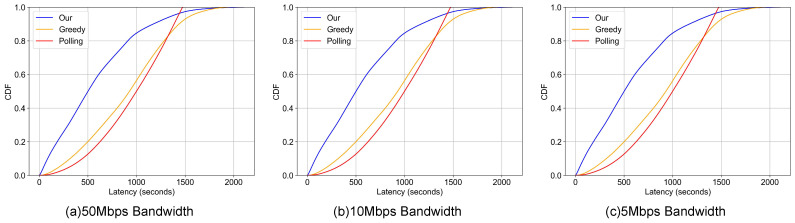
Anomaly End-to-End Latency Comparison. *For different gateway uplink bandwidth.*

This section presents a comprehensive analysis of the end-to-end latency. For the purpose of this evaluation, this metric is strictly defined as the aggregate of queueing delay (waiting time in both node and gateway buffers) and transmission delay (serialization time based on link bandwidth). Propagation delay is omitted as it is negligible relative to the serialization time in low-bandwidth IoT environments, and post-reception application processing time is excluded to isolate and quantify the efficiency of the proposed communication architecture. The analysis is supported by data from Table 1 (overall latency) and Table 2 (anomalous data latency), with corresponding visualizations in Figure 5 and Figure 6.

Across all tested bandwidth conditions, our dynamic priority-based scheme consistently outperforms both the conventional polling and greedy schemes in reducing latency for both overall and anomalous data. This performance advantage is particularly notable in the 10 Mbps bandwidth scenario where at the P50 metric for all data our scheme achieves a latency of 501.080 s, which is a significant reduction compared to the polling and greedy approaches. This trend continues at the P90 metric, where our scheme’s latency of 1170.770 s again surpasses the baselines. These performance gains are directly attributable to our method’s core design: dynamically prioritizing the transmission of anomalous data and adaptively allocating network resources to affected photovoltaic nodes, thereby enhancing overall system responsiveness.

Crucially, the proposed scheme demonstrates significant gains in Tail Latency (P90 and P95), which serves as the primary indicator for system reliability in safety-critical applications. As detailed in Table 2 (10 Mbps scenario), our method achieves a P90 latency for anomalous data of 1170.77 s, outperforming both the conventional polling scheme (1386.24 s) and the greedy scheme (1438.07 s). This represents a reduction of approximately 15.5% to 18.6% in the 90th percentile latency. The trend persists at the P95 level, where our approach (1373.61 s) continues to lead the baselines. This result confirms that dynamic prioritization does not merely improve average performance by sacrificing outliers; rather, it effectively shifts the entire latency distribution for fault data, ensuring that 95% of critical anomalies are delivered faster than they would be under the rigid constraints of traditional cyclic polling. While the deterministic nature of conventional polling provides a tightly bounded P99, our approach offers a superior trade-off by drastically reducing the detection time for the vast majority of faults.

In contrast, the baseline schemes exhibit significant limitations. While the greedy scheme shows marginal advantages over polling in select, low-load cases, its performance degrades substantially under heavy traffic. The accumulation of prolonged data queues at the nodes leads to delayed polling cycles and increased latency. The conventional polling method, however, delivers more consistent and competitive latency in certain contexts. For example, as shown in Table 1, its ability to ensure uniform and regular data transmission allows it to maintain a reasonable latency profile across all metrics, preventing the extreme delays seen with the greedy scheme under congestion.

The Cumulative Distribution Function (CDF) curves in Figure 5 and Figure 6 further corroborate these findings. For overall data latency (Figure 5), the curve for our scheme is distinctly shifted to the left of the baseline curves, particularly within the lower latency range (e.g., <1000 s). This shift indicates a lower probability of experiencing high latency compared to the polling and greedy methods in typical operational conditions. While the curves converge at the extreme high-latency tail, suggesting comparable performance in worst-case scenarios, the advantage in the primary operational range is clear.

This performance differentiation is even more pronounced for anomaly-specific latency (Figure 6). Here, our scheme’s CDF curve demonstrates a significant and sustained shift to the left across a much wider latency spectrum. This pattern underscores our approach’s superior capability to mitigate delays for critical, fault-related data, maintaining a lower probability of high latency even as overall system delays increase. This result directly aligns with our design principle of priority-based resource allocation, which effectively minimizes bottlenecks for time-sensitive anomalous data flows.

In summary, our dynamic priority scheme demonstrates superior end-to-end latency reduction by intelligently managing both routine and anomalous data flows. While conventional polling provides stability through uniform scheduling and the greedy approach shows situational advantages under light loads, our scheme delivers robust and balanced performance across all tested scenarios, establishing it as a more effective solution for modern power communication network applications. It is important to note that the significant reduction in tail latency (P99) observed in Table 1 also serves as a critical indicator of network congestion and system stability. In the conventional greedy scheme, the high P99 values imply that data packets frequently accumulate in gateway buffers, leading to extended queue lengths and a higher risk of buffer overflow. In contrast, our dynamic priority scheme effectively maintains a near-zero queue length for anomalous data, which means that such critical data is transmitted immediately upon reaching the gateway without accumulation. By keeping the critical data buffers clear, the system demonstrates superior stability and is less susceptible to cascading congestion, even under bandwidth-constrained scenarios (e.g., 5 Mbps) where traditional methods struggle.

### 5.2. Case Study: Automated Diagnostic Analysis of Panel 959

To demonstrate the practical application and diagnostic acuity of our DeepSeek-based system, this section presents a detailed case study of an automated analysis performed for a single entity, Panel 959. The analysis covers a midday period on the simulated date of 11 January 2021, and showcases how the system integrates environmental context, real-time data, and historical trends to generate a comprehensive diagnostic report.

#### 5.2.1. Environmental and Operational Context

The system first established the operational context for the analysis period (12:02–12:43):

**Meteorological Conditions.** The environment was characterized by Winter season conditions, with clear skies (0.0% cloud cover) and ambient temperatures just above freezing (0.65–0.69 °C).

**Real-Time Performance.** Over three consecutive readings, the panel’s actual power output (ranging from 69.50 W to 71.53 W) was observed to be stable and correctly correlated with the fluctuating solar irradiance (ranging from 505.34 W/m^2^ to 544.86 W/m^2^). In all instances, the power output exceeded the calculated theoretical minimum, indicating no significant power conversion inefficiency.

**Historical Trends.** An analysis of the 15 most recent data records (12:24–12:43) confirmed stable performance, with both irradiance and power output exhibiting only minor fluctuations within normal operational bounds. This allowed the system to rule out gradual panel degradation or intermittent performance drops.

#### 5.2.2. Anomaly Detection and Severity Assessment

Based on the contextual analysis, the system identified one primary anomaly:

**Anomaly.** Missing windSpeed Sensor Data.

**Severity.** Assessed as Low to Medium.

**Justification.** While not directly impacting the panel’s immediate power generation, the system reasoned that the complete absence of this data across all monitoring instances obstructs the evaluation of critical environmental factors, such as wind-driven panel cooling dynamics or natural snow-clearing processes.

#### 5.2.3. Root Cause Inference

The system then performed a root cause analysis for two distinct observations:

**Minor Irradiance Deficit.** Although power conversion was healthy, the measured irradiance was slightly below the theoretical maximum for clear sky conditions. The system correlated this observation with the sub-1 °C temperatures and inferred the most probable cause to be light snow or frost accumulation on the panel surface. It further hypothesized that the lack of wind (indicated by the missing windSpeed data) may have exacerbated this condition by preventing natural clearing of the panel.

**Missing Wind Data.** The system deduced that the sensor issue was isolated, as other environmental sensors were reporting normally. The most probable causes were identified as either sensor failure due to extreme cold (e.g., a frozen anemometer) or a localized communication disruption in the sensor’s network path.

#### 5.2.4. Maintenance Recommendations

Finally, the system generated a prioritized and actionable set of recommendations for operational personnel:

**Inspection.** Physically inspect Panel 959 for snow or frost accumulation and clean the surface if necessary. Verify that the panel’s tilt angle is optimized for winter conditions.

**Corrective Maintenance.** Investigate the windSpeed sensor’s power supply and network connectivity. Attempt to reboot or recalibrate the unit; replace it if it remains unresponsive.

**Preventive and Long-Term Actions.** To prevent recurrence, consider installing heating elements or applying anti-icing coatings to panels. Evaluate the use of heated enclosures for critical meteorological sensors in cold climates.

**Proactive Monitoring.** Schedule a follow-up IV curve trace to confirm the panel’s long-term electrical health. Increase the frequency of winter inspections to every 72 h until the issue is confirmed resolved. If power anomalies persist post-maintenance, validate the calibration of the irradiance sensor.

This case study exemplifies the system’s ability to move beyond simple data reporting to provide a multi-layered, context-aware diagnostic analysis, distinguishing between performance-critical issues and ancillary sensor faults while generating targeted, actionable intelligence.

### 5.3. Practical Feasibility Analysis

We assess the real-world industrial viability of the proposed framework across three critical dimensions, namely hardware requirements, operational expenditure, and deployment architecture trade-offs:

**Hardware Requirements.** The architecture intentionally minimizes on-site hardware costs. Edge nodes, i.e., DPV sensors, require only low-power industrial microcontrollers such as STM32 or ESP32 series, capable of performing simple threshold comparisons and managing standard TCP/IP stacks, yielding a per-unit cost below $5. On the server side, unlike conventional private cloud deployments that necessitate costly GPU clusters exemplified by NVIDIA A100, our framework integrates third-party Application Programming Interface (API) services for Large Language Models. This eliminates capital expenditure on dedicated computing infrastructure, enabling the central server to operate as a lightweight API gateway.

**Operational Costs.** The inference cost follows a token-based pricing model that proves highly economical for this application domain. First, the event-driven design triggers LLM inference only upon anomaly detection, thereby filtering out over 90% of routine monitoring traffic. Second, diagnostic prompts contain substantial static content, including environmental rules, panel metadata, and output formatting templates, which the system leverages through context caching. This reduces input costs to approximately $0.03 per million tokens. Consequently, even with a 4K-token context window per diagnosis, the marginal cost per fault analysis remains below $0.001, substantially lower than potential revenue loss from undetected failures or manual site inspections.

**Deployment Architecture Trade-offs.** While edge-native AI remains an active research area, deploying LLMs on resource-constrained DPV nodes with power budgets below 1 W is presently impractical due to thermal and energy constraints. Our hybrid architecture optimizes this trade-off, utilizing low-latency edge processing for immediate priority scheduling and traffic filtering, while harnessing the scalable reasoning capacity of cloud-based APIs for complex diagnostic tasks.

While the cloud handles complex reasoning, the edge remains efficient. Table 3 provides a consolidated comparison of these operational trade-offs, summarizing the communication, diagnostic, and computational characteristics of our proposed framework against traditional industrial baselines.

### 5.4. Parameter Sensitivity Analysis

To rigorously validate the rationale behind setting the recovery threshold to three consecutive transmissions (K = 3), we conduct a parameter sensitivity analysis. The experimental environment is configured with 5000 distributed nodes managed by 100 regional gateways, operating under a constrained uplink bandwidth of 10 Mbps. To capture the steady-state queueing behavior and latency distribution, each simulation scenario (K∈{1,2,3,4,5}) is executed for a duration of 5 min. This duration is selected to allow sufficient time for the priority queues to stabilize and for potential congestion patterns to manifest.

**Figure 7:**
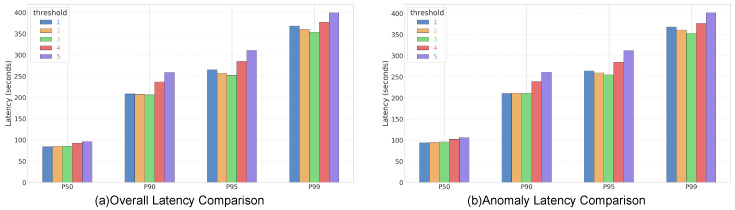
Parameter sensitivity analysis of the recovery threshold (K) under a 10 Mbps bandwidth constraint. (**a**) Comparison of Overall Latency across different percentiles. (**b**) Comparison of Anomaly-specific Latency.

Figure 7 illustrates the impact of the threshold setting on End-to-End Latency. The results indicate a clear trade-off between system stability and resource availability. When the threshold is set too low (K = 1, 2), the system is overly sensitive, leading to state oscillation where nodes frequently downgrade and re-upgrade their priority status, resulting in increased scheduling overhead and higher P90 latency. Conversely, when the threshold is overly conservative (K = 4, 5), nodes retain the anomalous high-priority status long after recovering. This behavior unnecessarily consumes scarce bandwidth, causing congestion that significantly spikes the P99 latency for other critical data. The threshold of K = 3 achieves the optimal balance, exhibiting the lowest latency across the upper percentiles P90 to P99 by effectively filtering transient noise without inducing network saturation.

## 6. Conclusions

This paper presents a holistic solution to the challenges of data scheduling and fault diagnosis in large-scale DPV systems. Through rigorous simulation, we demonstrated that our novel dynamic priority-based scheduling scheme significantly improves the timely delivery of critical fault data compared to conventional methods, especially under bandwidth constraints. This marked improvement, which stems from dynamically elevating data priority, is most pronounced in median latency (P50), with long-tail latency presenting an avenue for future optimization. Complementing this, our integration of the DeepSeek LLM agent automates diagnostics, providing actionable, expert-level insights by synthesizing environmental, historical, and real-time data. These two contributions, a responsive scheduling mechanism and an intelligent diagnostic agent, collectively provide a robust framework for efficient and reliable DPV operation. Future efforts will be directed at enhancing long-tail latency performance and incorporating predictive maintenance capabilities. Furthermore, we plan to explore advanced data augmentation strategies, specifically the utility of ‘positive-incentive noise’ in AI training. While this study focused on mitigating transmission noise, future iterations of the diagnostic agent could leverage controlled noise injection to improve model robustness and generalization, transforming environmental stochasticity from a disturbance into a feature for enhancing fault pattern recognition.

## Figures and Tables

**Table 1:** Overall End-to-End Latency. *Parentheses show % better than conventional greedy scheme.*

Metric	Latency (s)								
	50 Mbps			10 Mbps			5 Mbps		
	Our	Polling	Greedy	Our	Polling	Greedy	Our	Polling	Greedy
P50	**644.206**	737.327	683.391	**644.279**	737.328	684.316	**644.388**	737.328	685.440
	(5.73%)	(−7.89%)	(0.00%)	(5.85%)	(−7.75%)	(0.00%)	(5.99%)	(−7.57%)	(0.00%)
P90	1437.384	**1325.396**	1373.895	1437.430	**1325.397**	1374.965	1437.507	**1325.397**	1376.278
	(−4.62%)	(3.53%)	(0.00%)	(−4.54%)	(3.61%)	(0.00%)	(−4.45%)	(3.70%)	(0.00%)
P95	1604.633	**1398.929**	1512.931	1604.671	**1398.930**	1513.990	1604.739	**1398.930**	1515.318
	(−6.06%)	(7.54%)	(0.00%)	(−5.99%)	(7.60%)	(0.00%)	(−5.90%)	(7.68%)	(0.00%)
P99	1862.179	**1457.838**	1759.333	1862.216	**1457.839**	1760.411	1862.290	**1457.839**	1761.734
	(−5.85%)	(17.14%)	(0.00%)	(−5.78%)	(17.19%)	(0.00%)	(−5.71%)	(17.25%)	(0.00%)

Note: Bold values indicate the lowest latency for each percentile.

**Table 2:** Anomaly End-to-End Latency. *Parentheses show % better than conventional greedy scheme.*

Metric	Latency (s)								
	50 Mbps			10 Mbps			5 Mbps		
	Our	Polling	Greedy	Our	Polling	Greedy	Our	Polling	Greedy
P50	**501.035**	999.618	928.130	**501.080**	999.618	929.232	**501.163**	999.619	930.566
	(46.02%)	(−7.70%)	(0.00%)	(46.08%)	(−7.57%)	(0.00%)	(46.14%)	(−7.42%)	(0.00%)
P90	**1170.695**	1386.238	1437.009	**1170.770**	1386.238	1438.072	**1170.854**	1386.239	1439.474
	(18.53%)	(3.53%)	(0.00%)	(18.59%)	(3.60%)	(0.00%)	(18.66%)	(3.70%)	(0.00%)
P95	**1373.558**	1429.427	1561.892	**1373.612**	1429.427	1562.997	**1373.655**	1429.428	1564.342
	(12.06%)	(8.48%)	(0.00%)	(12.12%)	(8.55%)	(0.00%)	(12.19%)	(8.62%)	(0.00%)
P99	1691.475	**1463.977**	1784.102	1691.539	**1463.978**	1785.182	1691.599	**1463.978**	1786.610
	(5.19%)	(17.94%)	(0.00%)	(5.25%)	(17.99%)	(0.00%)	(5.32%)	(18.06%)	(0.00%)

Note: Bold values indicate the lowest latency for each percentile.

**Table 3:** Comparative Summary.

Metric	Conventional Polling	Conventional Greedy	Proposed Framework
Communication Performance	High latency. Linear scaling; faults delayed by cycle.	Unstable. Low latency at light load; severe jitter under congestion.	Optimized. Priority reduces P50 latency by ∼49%; ensures reliable delivery.
Diagnostic Performance	None. Raw data only; manual analysis required.	None. Raw data only; manual analysis required.	Intelligent. Zero-shot, explainable; automates root cause analysis.
Computational Cost	Negligible. Minimal MCU overhead.	Negligible. Pure transmission; minimal processing.	Hybrid. Lightweight edge & cost-effective cloud API via caching.

## Data Availability

The original contributions presented in this study are included in the article. Further inquiries can be directed to the corresponding author.

## Abbreviations

The following abbreviations are used in this manuscript:

RF	Radio Frequency
DPV	Distributed Photovoltaic
EMI	Electromagnetic Interference
LLM	Large Language Model
ML	Machine Learning
SVM	Support Vector Machine
IoT	Internet of Things
WSN	Wireless Sensor Network
FIFO	First-In-First-Out
QoS	Quality of Service
DC	Direct Current
AC	Alternating Current
DAS	Data Acquisition System
LPWAN	Low-Power Wide-Area Network
MoE	Mixture-of-Experts
NTP	Network Time Protocol
P50	50th Percentile Latency (Median)
P90	90th Percentile Latency
P95	95th Percentile Latency
P99	99th Percentile Latency
API	Application Programming Interface
